# miRNA-132-3p inhibits osteoblast differentiation by targeting Ep300 in simulated microgravity

**DOI:** 10.1038/srep18655

**Published:** 2015-12-21

**Authors:** Zebing Hu, Yixuan Wang, Zhongyang Sun, Han Wang, Hua Zhou, Lianchang Zhang, Shu Zhang, Xinsheng Cao

**Affiliations:** 1 The Key Laboratory of Aerospace Medicine, Ministry of Education, The Fourth Military Medical University, 710032, Xi’an, Shaanxi, China; 2 Department of orthopedics, No.454 Hospital of PLA, Nanjing, Jiangsu 210002, P.R. China

## Abstract

Recent studies have demonstrated that miRNAs can play important roles in osteoblast differentiation and bone formation. However, the function of miRNAs in bone loss induced by microgravity remains unclear. In this study, we investigated the differentially expressed miRNAs in both the femur tissues of hindlimb unloading rats and primary rat osteoblasts (prOB) exposed to simulated microgravity. Specifically, miR-132-3p was found up-regulated and negatively correlated with osteoblast differentiation. Overexpression of miR-132-3p significantly inhibited prOB differentiation, whereas inhibition of miR-132-3p function yielded an opposite effect. Furthermore, silencing of miR-132-3p expression effectively attenuated the negative effects of simulated microgravity on prOB differentiation. Further experiments confirmed that E1A binding protein p300 (Ep300), a type of histone acetyltransferase important for Runx2 activity and stability, was a direct target of miR-132-3p. Up-regulation of miR-132-3p by simulated microgravity could inhibit osteoblast differentiation in part by decreasing Ep300 protein expression, which, in turn, resulted in suppression of the activity and acetylation of Runx2, a key regulatory factor of osteoblast differentiation. Taken together, our findings are the first to demonstrate that miR-132-3p can inhibit osteoblast differentiation and participate in the regulation of bone loss induced by simulated microgravity, suggesting a potential target for counteracting decreases in bone formation.

Numerous studies have shown that mechanical stimulations play an important role in the maintenance of bone homeostasis, skeletal morphology and strength during bone formation and development^1^,^2^,^3^. By contrast, skeletal unloading, as observed in space flight astronauts or in patients subjected to prolonged immobility or bed-rest, typically induces severe bone loss^4^. The early studies described similar phenomenon, such as cancellous osteoporosis in weight-bearing bones, decreased bone formation and abnormal bone metabolism after space flight^5^,^6^. During the spaceflight mission on the Soviet/Russian MIR spacecraft and the International Space Station, crew members experienced a persistently enhanced areal bone mineral density lost at an average monthly rate of 1.06% from the spine and 1.0 to 1.6% from the hip, despite adopting an intense exercise regimen to counteract mechanical unloading^7^. Decreased bone formation in both rat cortical and cancellous bones was also demonstrated by tetracycline labeling before and after space flight^8^,^9^,^10^,^11^. In view of spaceflight tremendous costs, more studies have been performed on the ground. The hindlimb unloading (HU) model is a well-tolerated method to mimic the cephalic fluid shift and removal of skeletal weight-bearing loads seen in spaceflight^12^. Despite the variability of data among independent studies, this model successfully replicates an osteopenia characterized by decreased bone mineral content, weakened bone resistance, and loss of femoral mass, similar to that observed in spaceflight data^13^,^14^. Moreover, cell-based studies have also been performed using rotational devices, such as the Rotating Wall Vessel (RWV) or Random Positioning Machine (RPM) systems. These devices constantly rotate around at least one axis to produce a vector-averaged gravity so that cells are unable to sense gravity^15^,^16^. Exposure to such rotational systems can significantly inhibit the differentiation and mineralization of osteoblasts while increasing the differentiation of osteoclast-like cells^17^,^18^, effects that are similar to those of microgravity on bone cells.

The mechanism of bone loss induced by microgravity has not yet been clearly elucidated. One point many studies have agreed upon is that abnormal osteoblast function and development are the main reasons for microgravity-induced bone loss^19^,^20^,^21^. Studies have demonstrated that the development of osteoblasts is markedly affected when exposed to real or simulated microgravity conditions. In a ground-based, simulated microgravity environment, human mesenchymal stem cells, multipotent cells that can differentiate into several lineages of mesenchymal tissues including bone, cartilage, fat and muscle, were accelerated to differentiate along the adipocyte lineage, whereas the osteoblast lineage was inhibited^17^. During a four day space flight experiment, the flown osteoblasts grew more slowly and had lower growth responsiveness to serum stimulation than those on the ground. The cytoskeleton of the flight osteoblasts had fewer stress fibers, unique abnormal morphology and 30% smaller nuclei than the ground group^22^. Several osteoblast markers, such as alkaline phosphatase (ALP), the runt-related transcription factor 2 (Runx2) and Osteocalcin, were suppressed after exposing MC3T3-E1 osteoblasts to RWV for 24 h^23^. However, how the development of osteoblasts is regulated during microgravity exposure remains unclear.

miRNAs are small non-coding RNAs ~22 nucleotides long that can participate in broad biological processes by elaborately regulating gene expression^24^. The application of microarray technologies can facilitate the expression profiling of miRNAs in many different tissues and cells because of its high sensitivity, throughout and comparative capabilities^25^. Recently, several studies identified populations of miRNAs during osteoblast differentiation using microarray analysis^26^,^27^. miR-27 can promote osteoblast differentiation through modulation of Wnt signaling by targeting Apc genes^28^. Enhanced Wnt signaling further activates the expression of miR-34, another promoter of osteoblast differentiation involved in the regulation of Notch signaling, resulting in a sophisticated cascade regulatory network^29^. Osterix (Osx), a zinc finger transcription factor and critical regulator of osteoblast mineralization, was inversely correlated with miR-93, indicating a novel miR-93/Osx regulatory feedback loop in osteoblast mineralization^30^. Our group demonstrated that miR-103-3p inhibited MC3T3-E1 osteoblast-like cell proliferation mainly by suppressing the expression of Cav1.2 protein, the primary subunit of L-type voltage sensitive calcium channels^31^. In addition, the expression of several important regulators, such as Runx2, BMP2, SATB2, and TGF-β, are controlled by different miRNAs during the multistep processes of osteoblast differentiation^32^,^33^. miRNAs that serve as negative regulators have also been identified in bone formation. Overexpression of miR-182 in osteoblast lineage cells increases cell apoptosis and hinders osteoblast proliferation and differentiation by inhibiting the expression of FoxO1^34^. All of these studies suggest that miRNAs play an important role in the regulation of osteoblast differentiation and bone formation. However, few studies have investigated the role of miRNAs in the regulation of osteoblast function under a microgravity environment.

In this study, we screened for differentially expressed miRNAs in rat femur tissue after 3 weeks of HU using microarray profiling. Then parts of miRNAs were submitted for further verification and functional analysis in prOB cells cultured in simulated microgravity environment. Specially, miR-132-3p was found up-regulated and negatively correlated with osteoblast differentiation and bone formation. By suppressing the protein translation process of Ep300, an important transcriptional co-activator and histone acetyltransferase, miR-132-3p could significantly decrease Ep300-activited Runx2 activity and acetylation, resulting in the inhibition of osteoblast differentiation. Further studies showed that therapeutic inhibition of miR-132-3p in osteoblasts resulted in a decrease of Ep300 expression, effectively blocking the negative effect of simulated microgravity on osteoblast differentiation *in vitro*.

## Results

### Bone loss induced by unloading correlates with alteration of miRNA expression in rat femurs

**Figure 1:**
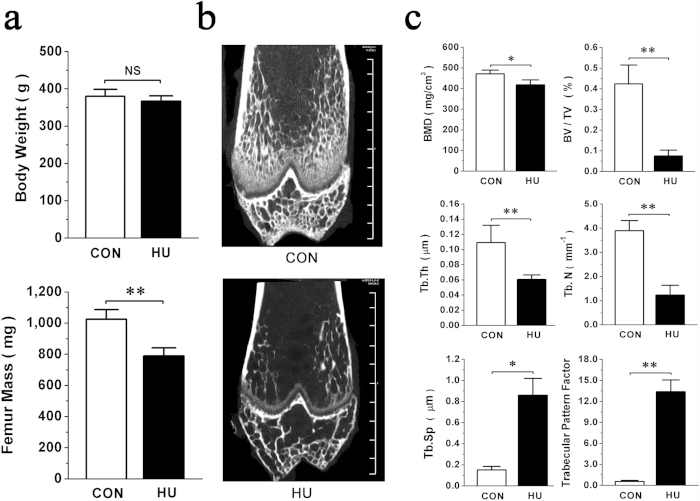
Hind-limb unloading significantly inhibits bone formation *in vivo*. (**a**) Body weight and femur mass in Hind-limb Unloading (HU) and pair-fed control (CON) rats. (**b**) Representative images illustrating the effects of unloading on the bone mass of the distal femur of CON and HU rats by micro-CT analysis. Scale bars, 1 mm. (**c**) Three-dimensional microstructure parameters of distal femurs from CON and HU rats, including bone mineral density (BMD), relative bone volume (BV/TV), trabecular thickness (Tb.Th), trabecular number (Tb.N), trabecular space (Tb.Sp), and trabecular pattern factor. All data are provided as means ± SD. For each group n = 8, *P < 0.05, **P < 0.01. NS, not significant.

Hindlimb unloaded (HU) rats were selected for a simulated microgravity-induced bone loss model and monitored by micro-CT examination. The non-significant difference observed between control (CON) and HU body weights suggested that the stress caused by tail suspension was well tolerated by the rats. The dramatic decrease of fat-free femur mass in HU group after unloading for 3 weeks demonstrated the success of this model (Fig. 1a). The HU femurs presented with a typical osteopenia phenotype which was characterized by sparse, fractured and inconsecutive trabecular architecture (Fig. 1b). Three-dimensional architecture parameters also showed corresponding changes with significant decreases in bone mineral density (BMD), relative bone volume (BV/TV), trabecular thickness (Tb.Th) and trabecular number (Tb.N) and remarkable increases in trabecular separation (Tb.Sp) and trabecular pattern factor (TPF) (Fig. 1c).

**Figure 2:**
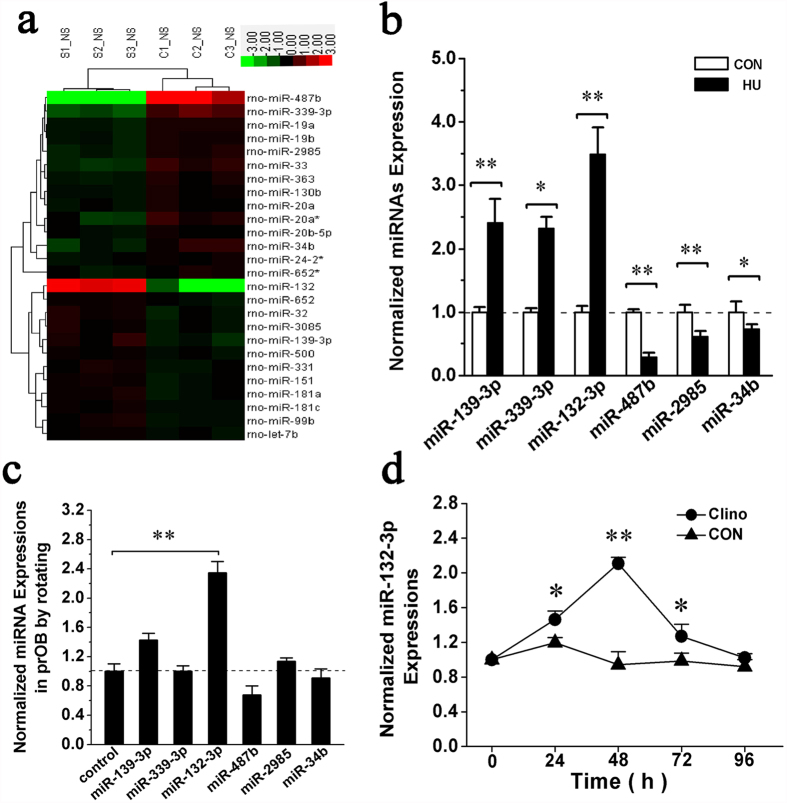
Up-regulation of miR-132-3p both in the bone tissue of HU rat femurs and prOB cells cultured in simulated microgravity. (**a**) Alterations of miRNA expression in the femur bone tissue of CON and HU rats examined by Agilent miRNA arrays. Green represents down-regulated miRNAs, and red represents up-regulated miRNAs, with the color scale in the upper right corner indicating the relative expression levels. (**b**) qRT-PCR analysis of miR-139-3p, -339-3p, -132-3p, -487b, -2985 and -34b levels partly selected from the array data (normalized to internal reference U6). (**c**) Expression levels of miR-139-3p, -339-3p, -132-3p, -487b, -2985 and -34b in prOB cells after exposure to clinorotation (Clino) for 48 h examined by qRT-PCR. The relative ratio is shown with that of cells under static control conditions. (**d**) Time course of miR-132-3p expression in prOB cells after exposure to clinorotation. Fold-increase is provided in comparison to the static control group. For each group, values are mean ± SD, n = 3, *P < 0.05, **P < 0.01.

To test the hypothesis that miRNA expression of bone tissue is altered during bone loss induced by simulated microgravity, we performed miRNAs microarray profiling of femur bone tissues from the HU and CON rats. Among the 678 miRNA probes evaluated, expression levels of only 25 were significantly altered, with 11 miRNAs up-regulated and 14 miRNAs down-regulated in femurs of HU rats compared with that of the CON groups (Fig. 2a). Some miRNAs were selected for validation by quantitative RT-PCR (qRT-PCR) based on their fold changes. qRT-PCR analysis showed that the expression levels of miR-139-3p,-339-3p and -132-3p were up-regulated, and the expression levels of miR-487b, -2985 and -34b were down-regulated during bone loss (Fig. 2b).

### Exposure to the clinostat up-regulates miR-132-3p expression levels in osteoblasts *in vitro*

To further validate whether the differentially expressed miRNAs keep the corresponding variation in osteoblasts exposed to simulated microgravity conditions, prOB cells were cultured in a Rotating Wall Vessel Bioreactor (RWVB) clinostat and tested after rotating for 48 h. The effects of clinorotation were assessed by observing ALP activity and expression level of Runx2, the transcription factors critical for osteoblast differentiation (data not shown). Results of qRT-PCR showed that expression of miR-132-3p was significantly up-regulated compared with other miRNAs during clinorotation (Fig. 2c), displaying a similar variation in bone tissue. Next, a further experiment revealed the time-dependent variation in miR-132-3p expression which was kept a continuous increase upon rotating and peaked at 48 h and then subsequently decreased close to the normal level during a 96 h clinorotation (Fig. 2d).

### miR-132-3p inhibits osteoblast differentiation *in vitro*

**Figure 3:**
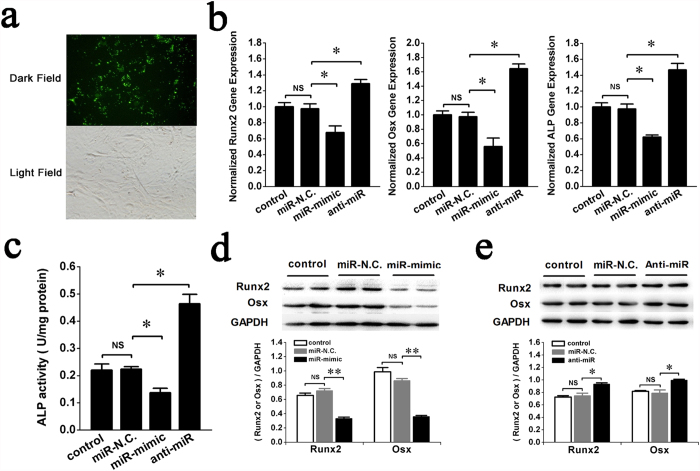
miR-132-3p inhibits osteoblast differentiation *in vitro*. To study the effects of miR-132-3p on osteoblast differentiation, prOB cells were transfected with either a miR-132-3p mimic (miR-mimic, 60 nM), an inhibitor (anti-miR, 80 nM) or its homologous miRNA negative control (miR-N.C.). (**a**) Representative fluorescent images of prOB cells transfected with a miRNA nucleoside analogue for 24 h. The upper panel shows fluorescence in a dark field, and the lower panel shows the same cells in a bright field (original magnification 200×). (**b,c**) Osteoblast differentiation was confirmed by qRT-PCR analysis of osteoblast marker genes (Runx2, Osx and ALP normalized to GAPDH) and activity analysis of the ALP protein at 48 h. (n = 3). (**d,e**) Western blot analyses of Runx2 and Osx protein expression were performed and quantified using ImageJ software. (n = 4). For each group, values are mean ± SD, *P < 0.05, **P < 0.01. NS, not significant.

To evaluate the biological effects of miR-132-3p on osteoblast differentiation, the synthetic miR-mimic (analogue) and anti-miR (inhibitor) of miR-132-3p were transiently transfected into prOB cells to alter intracellular levels of miR-132-3p *in vitro* (Fig. 3a). The effects of those analogue and inhibitor on osteoblast differentiation were examined by observing the expression levels of the osteoblast-specific markers Runx2, Osx and ALP. qRT-PCR data showed that the gene expression levels of Runx2, Osx and ALP decreased following transfection of miR-mimic compared with transfection of miR-N.C. (Fig. 3b, miR-mimic group). ALP activity (Fig. 3c miR-mimic group) and protein expression of Runx2 and Osx (Fig. 3d) also decreased, suggesting that overexpression of miR-132-3p inhibited osteoblast differentiation. By contrast, Runx2, Osx and ALP gene expression (Fig. 3b, anti-miR group), ALP protein activity (Fig. 3c, anti-miR group), and Runx2, Osx protein levels (Fig. 3e) were all increased when miR-132-3p was down-regulated by transfection of anti-miR, suggesting that inhibition of miR-132-3p could promote osteoblast differentiation. These results indicate that miR-132-3p is an inhibitor of osteoblast differentiation.

### Silencing of miR-132-3p expression partially attenuates the negative effects of simulated microgravity on osteoblast differentiation *in vitro*

**Figure 4:**
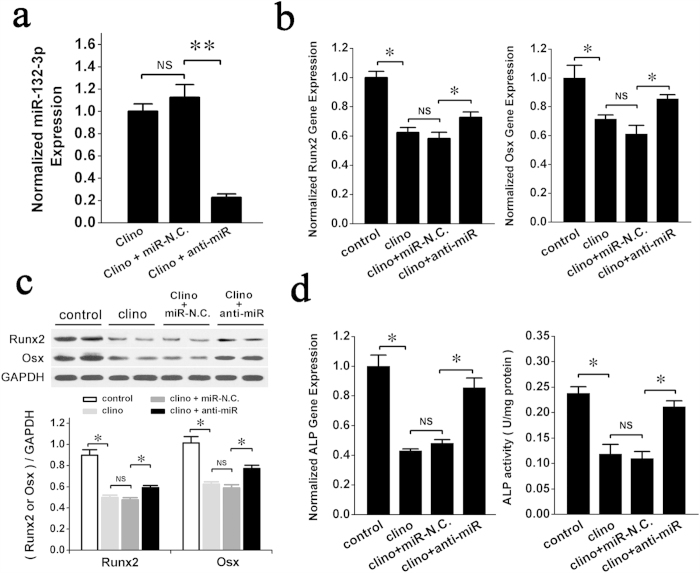
Down-regulation of endogenous miR-132-3p expression partly attenuates inhibition of osteoblast differentiation by clinorotation *in vitro.* prOB cells were transfected with miR-N.C. and anti-miR for 12 h and then exposed to clinorotation for 48 h. The relative parameter was detected. (**a**) miR-132-3p expression in prOB cells was analyzed by q RT-PCR. (n = 3). (**b**) Osteoblast differentiation was confirmed by qRT-PCR analysis of osteoblast marker genes (Runx2 and Osx normalized to GAPDH). (n = 3). (**c**) Western blot analysis of Runx2 and Osx protein expression was performed and quantified using ImageJ software. (n = 4). (**d**) ALP gene expression and protein activity were measured. (n = 3). For each group, values are mean ± SD, *P < 0.05, **P < 0.01. NS, not significant.

To test whether therapeutic inhibition of miR-132-3p could rescue the osteoblast differentiation decrease caused by simulated microgravity, prOB cells were transfected with the anti-miR of miR-132-3p for 12 h and then exposed to simulated microgravity for 48 h. miR-132-3p significantly decreased in the group transfected with anti-miR compared with the miR-N.C. group at the end of clinorotation (Fig. 4a). The gene expression levels and protein levels of differentiation markers, Runx2 and Osx, were increased in the anti-miR group (Fig. 4b,c). Meanwhile, ALP gene expression and protein activity were also increased in the anti-miR group (Fig. 4d). Our data show that down-regulation of endogenous miR-132-3p expression partially attenuates the inhibition of osteoblast differentiation by simulated microgravity *in vitro*. miR-132-3p may be a promising new therapeutic target to protect against the decreased bone formation induced by microgravity.

### miR-132-3p inhibits Ep300 protein expression in prOB cells exposed to a simulated microgravity environment

miRNAs exert their effects mainly through binding to the untranslated regions (UTRs) of target gene mRNAs^35^. To obtain further insight into the molecular mechanisms of the regulation mediated by miR-132-3p, we identified the potential targets of miR-132-3p that are related to osteoblast differentiation using the miRNA target prediction algorithms TargetScan and PicTar, which have low false positive rates. Among the target genes predicted by both algorithms, Ep300 was the most promising candidate for it ranked highly among the predicted genes and has been reported to function as an important regulator of cell differentiation.

**Figure 5:**
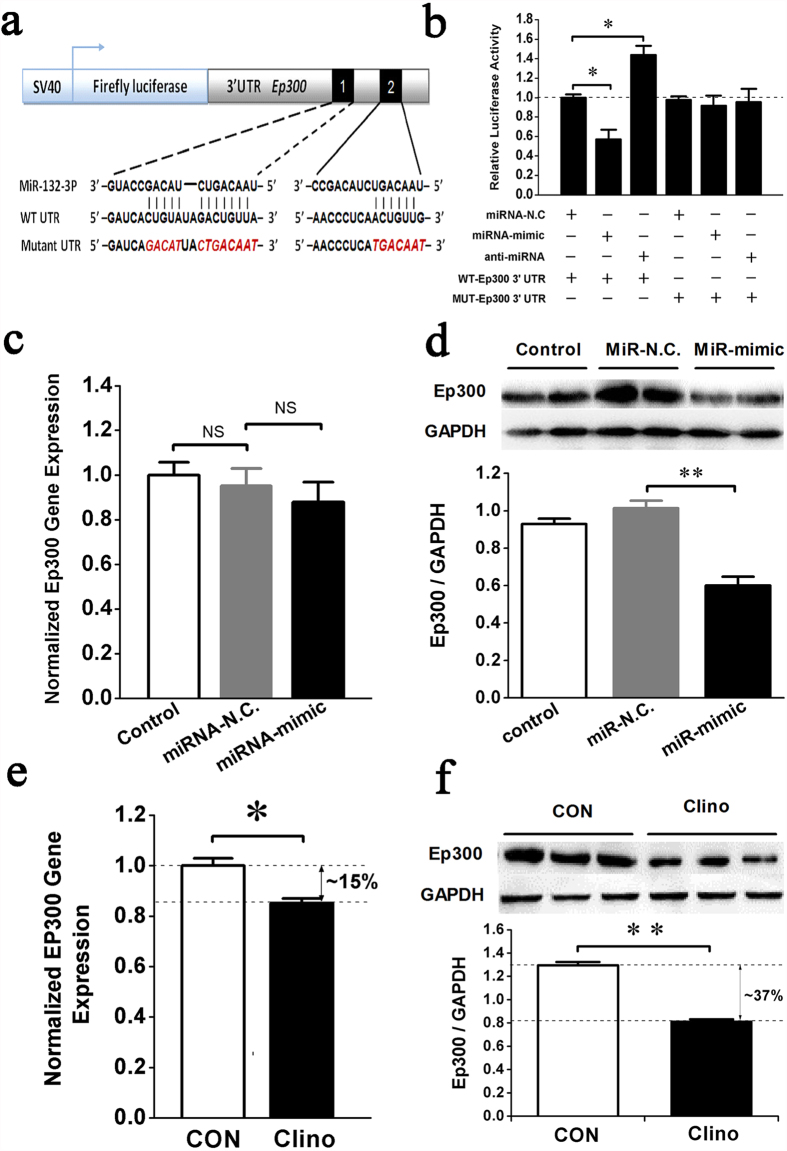
miR-132-3p directly inhibits Ep300 protein expression in prOB cells exposed to a simulated microgravity environment. (**a**) Schematic representation of luciferase constructs used for reporter assays. The two miR-132-3p target sites within the 3′ UTR of Ep300 are depicted as black boxes. Sequences below indicate putative miR-132-3p target sites on the wild type 3′ UTR, the mutated derivative, and the pairing regions of miR-132-3p. (**b**) The effect of the miR-132-3p mimic, the inhibitor or their negative controls on the luciferase activity of the WT Ep300 3′ UTR or the MUT Ep300 3′ UTR reporter in 293T cells. (n = 3). (**c,d**) Ep300 mRNA (n = 3) and protein expression (n = 4) in prOB cells were examined after transfection with miR-N.C. and miR-mimic for 48 h by qRT-PCR and western blot, respectively. (**e,f**) Ep300 mRNA and protein expression was detected by qRT-PCR and western blot, respectively, after exposure to clinorotation for 48 h. (n = 3). For each group, values are mean ± SD, *P < 0.05, **P < 0.01. NS, not significant.

To test whether miR-132-3p directly inhibits Ep300 protein translation by binding to a predicted target site in the 3′ UTR, a dual luciferase reporter system was constructed containing either the wild-type Ep300 3′ UTR sequence (WT) or an Ep300 3′ UTR mutant sequence (MUT) (Fig. 5a). The luciferase reporter assay showed that the miRNA-mimic of miR-132-3p decreased, but the anti-miRNA increased, the WT Ep300 3′ UTR luciferase reporter activity but not the MUT Ep300 3′ UTR reporter. By comparison, miRNA-N.C. had no effect on the luciferase activity when co-transfected with either the Ep300 3′ UTR or the Ep300 3′ UTR mutant (Fig. 5b). This result suggests that Ep300 is a direct target of miR-132-3p. Further testing demonstrated that miR-132-3p mainly suppressed protein translation of Ep300 (Fig. 5d) but had little effect on gene expression (Fig. 5c). Indeed, we observed a greater degree of change in the expression levels of the Ep300 protein (decreased approximately 37%, Fig. 5f) than of genes (decreased approximately 15%, Fig. 5e) after exposure of prOB cells to clinorotation for 48 h. It is possible, however, that the decrease in Ep300 gene expression we observed may involve some other mechanism and requires further investigation.

### Inhibition of Ep300 expression by miR-132-3p significantly decreases the stability and acetylation levels of Runx2

**Figure 6:**
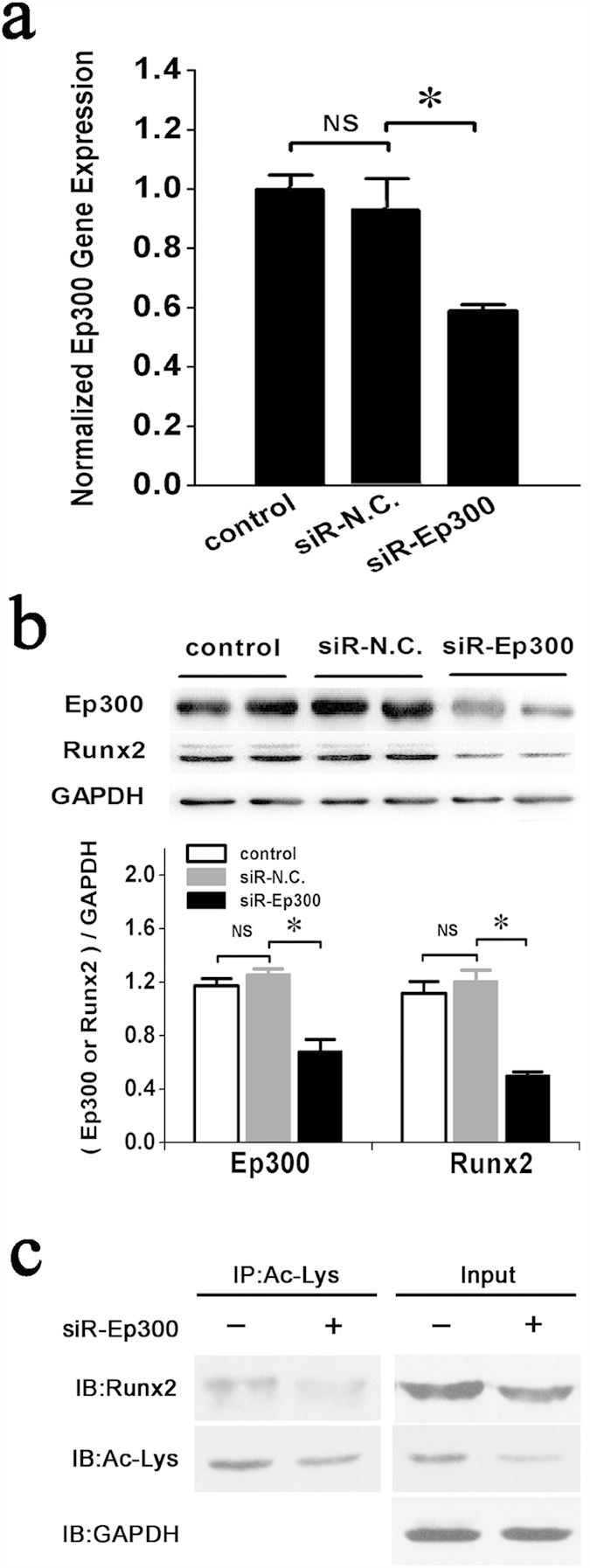
Inhibition of Ep300 expression by miR-132-3p decreases the stability and acetylation levels of Runx2. (**a**) qRT-PCR analysis of Ep300 gene expression in prOB cells was performed after transfection with siRNA-Ep300. (n = 3). (**b**) Western blot examination of Runx2 protein expression in prOB cells in which Ep300 expression was inhibited by siRNA. (n = 4). (**c**) Levels of acetylated Runx2 were detected by immunoprecipitation using an anti-acetyl-lysine antibody (Ac-lys), followed by a western blot with an anti-Runx2 antibody. (n = 3). For each group, values are mean ± SD, *P < 0.05. NS, not significant.

Ep300 is a histone acetyltransferase that can add acetyl groups to lysine residues of histone or non-histone target proteins to protect them from ubiquitin-mediated proteolysis^36^. Previous reports have shown that BMP2 signaling can increase the transactivation activity and inhibit the Smurf1-mediated degradation of Runx2 by stimulating Ep300-mediated Runx2 acetylation in the C2C12 cell line^37^. Therefore, we hypothesized that the suppression of Ep300 expression by miR-132-3p in prOB cells could decrease the stability and acetylation levels of Runx2. To test this hypothesis, siRNAs were used to silence Ep300 expression in prOB cells (Fig. 6a). Next, the levels of total and acetylated Runx2 proteins were examined. Our data indicated that suppression of Ep300 expression resulted in the down-regulation of both total Runx2 (Fig. 6b) and acetylated Runx2 (Fig. 6c) protein levels in prOB cells compared with the control group. Thus, our results demonstrate that miR-132-3p directly targets Ep300 and inhibits osteoblast differentiation in part by decreasing Ep300 expression, which, in turn, leading to suppression of the synergistic activity and acetylation of Runx2.

## Discussion

Until now, the mechanism of microgravity-induced bone loss has not been elucidated. The present study demonstrates that miRNAs can participate in the regulation of bone loss under simulated microgravity conditions. Specifically, miR-132-3p was up-regulated by simulated microgravity and functioned as a negative regulator of osteoblast differentiation. Inhibition of miR-132-3p by anti-miR-132-3p effectively attenuated the negative effects of clinorotation on *in vitro* osteoblast differentiation. These findings suggest that miR-132-3p plays a pivotal role in bone loss induced by simulated microgravity and is therefore a promising candidate for new therapeutic strategies.

During spaceflight, bone loss occurs mainly due to the lack of mechanical signal stimulations. These mechanical stimulations can be classified as two categories. One is the gravity which can be considered as a non-contact force. And another is contact force including static gravity-induced weight bearing, ground reaction force, and dynamic loading generated by muscular contractions during locomotion. To truly simulate the microgravity condition, we choose two kinds of models, the HU rat model and cell clinorotation model. In line with previous studies^38^,^39^, the HU rats in our experiments exhibited dramatic deterioration of the femur bone microarchitecture, as shown by the micro-CT analysis. Based on this model, the femur tissue was chosen for miRNA array profiling, and 25 differentially expressed miRNAs were identified for the first time. Of the differentially expressed miRNAs, several miRNAs, such as miR-20a, -181a, -34b, have already been reported to be involved in bone formation and osteoblast function^40^,^41^,^42^, confirming the reliability of our data. This high throughput screening revealed the relationship between miRNA expression and bone loss *in vivo*, truly reflecting the comprehensive body changes in microgravity, including effects of the nervous system and humoral regulation. Expression levels of some miRNAs were then examined in prOB cells exposed to clinorotation. miR-132-3p was up-regulated both in bone tissue and prOB cells after simulated microgravity exposure, suggesting its possible regulatory effects on osteoblast function. Two recent studies have also demonstrated the regulatory roles of miRNAs in bone formation and osteoblast function under a simulated microgravity environment. Wang showed that osteoblast-specific down-regulation of miR-214 levels could promote bone formation in HU mice, one of the three models they used to research human aged osteoporosis^43^. Our group also identified another miRNA, miR-103-3p, that inhibited osteoblast proliferation and L-type calcium channel function mainly through suppressing Cav1.2 expression under a simulated microgravity environment^31^,^44^. To obtain a better understanding of miRNA-based regulatory mechanisms in microgravity, miR-132-3p was studied in more detail.

Previously, miR-132-3p has been shown to play an important role in neurological development, synaptic transmission, inflammation, angiogenesis and even cancer. In neural cells, miR-132-3p promotes neuronal outgrowth and sprouting by decreasing the levels of p250GAP, a GTPase activating protein linked to neuronal differentiation^45^. It is notable, however, that some reports have indicated that miR-132-3p functions as a negatively regulator of the differentiation of dopamine neurons^46^. In addition, angiogenic factors, such as VEGF and bFGF, can promote transcription of miR-132-3p in endothelial cells, which further silences p120RasGAP expression and active conformation to induce proliferation^47^. This angiogenic role could implicate miR-132-3p in the oncogenesis of cancers, such as chronic lymphoblastic leukemia^48^, osteosarcoma^49^, and breast cancer^50^. All of these studies indicate a complicated and comprehensive regulatory role for miR-132-3p in cell proliferation and differentiation. However, the effects of miR-132-3p on osteoblast differentiation, particularly under the simulated microgravity conditions we described in our study, have not been previously reported.

Runx2 (also known as Cbfa1) is frequently described as the master of osteoblast differentiation and is influenced by many key signaling pathways and transcription factors^51^,^52^. The homozygous Runx2^−/−^ mice have a complete deficiency of functional osteoblasts and do not survive because of lack of mineralized bone^53^. ALP activity is always used as an indicator of cell osteogenesis differentiation. Osx is a zinc finger transcription factor expressed in osteoblasts and required for bone differentiation and mineralization^54^. Our *in vitro* experiments demonstrated that up-regulation of miR-132-3p in prOB cells significantly promoted the expression levels of Runx2, ALP and Osx, whereas down-regulation of miR-132-3p had an opposite effect on these markers. These data strongly suggest that miR-132-3p has a suppressive effect on osteoblast differentiation. It can be concluded that simulated microgravity can inhibits osteoblast differentiation partly by up-regulating the expression of miR-132-3p. Moreover, additional experiments demonstrated that silencing miR-132-3p by anti-miR-132-3p effectively attenuated the negative effect of clinorotation on *in vitro* osteoblast differentiation.

To study the molecular mechanisms by which miR-132-3p regulates osteoblast differentiation, we predicted the potential targets of miR-132-3p by using miRNA target prediction software. Notably, the 3′ UTR of Ep300 possesses a 7-nt perfect match site for the miR-132-3p seed region predicted by both the TargetScan and PicTar algorithms. Ep300 has been reported to function as an important factor in the regulation of osteoblast differentiation^55^,^56^,^57^. Our experimental data demonstrated that miR-132-3p overexpression results in down-regulation of Ep300 at the protein level, whereas functional inhibition of miR-132-3p by its anti-miRNA results in an increase in Ep300, strongly suggesting that Ep300 is regulated by miR-132-3p. Indeed, our Ep300 3′ UTR luciferase reporter assay confirmed that Ep300 is a direct target of miR-132-3p. Previous studies have suggested that Ep300, a histone acetyltransferase, is capable of increasing the half-life of Runx2 and its transcriptional activity through stimulation of Runx2 acetylation in C2C12 cell lines^58^. Another study showed that Erk activation can enhance the stability and transcriptional activation of Runx2 by increasing Ep300 protein levels^57^, suggesting the possible involvement of Ep300 in the regulation of Runx2 expression. However, whether this function is maintained in the simulated microgravity-mediated regulation of osteogenesis remains unclear. In our study, overexpression of miR-132-3p decreased the transcriptional activity of Runx2 and was coordinated with the change during silencing of Ep300 expression by targeted siRNA. Thus, according to our studies and previous reports, up-regulation of miR-132-3p induced by simulated microgravity can decrease the acetylation and transcriptional activity of Runx2 by inhibiting expression of the histone acetyltransferase Ep300. The low levels of Runx2 attenuate the differentiation of osteoblasts and the expression of critical osteogenic factors.

It should be noted that there are some limitations to our study. We examined the function of miR-132-3p in prOB cell differentiation *in vitro* under simulated microgravity. However, the *in vivo* effects, such as whether silencing of miR-132-3p expression could counter the bone loss observed in HU rats, were not examined. Additionally, there are more than a hundred of predicted targets for miR-132-3p in TargetScan and PicTar algorithms. Whether these undisclosed target genes could participate in the miR-132-3p-mediated osteoblast differentiation is not known. These limitations should be addressed in future studies.

To our knowledge, this is the first report demonstrating that miR-132-3p serves as a negative regulator of the osteoblast differentiation in simulated microgravity by hindering translation of Ep300, which in turn, results in suppression of the synergistic activity and stability of Runx2. Importantly, our results demonstrate that functional inhibition of miR-132-3p can accelerate osteogenic differentiation and effectively attenuate the negative effect of clinorotation on *in vitro* osteoblast differentiation, suggesting that therapeutic approaches targeting miR-132-3p may be useful for enhancing bone formation and may be protective against microgravity-induced bone loss.

## Method

### Hindlimb Unloading Rat Model

Animal studies were performed using the HU model, which is considered an effective model of bone loss induced by weightlessness and has been described previously^13^. Briefly, Male Sprague-Dawley (SD) rats at 7w age were individually caged and suspended by the tail using a strip of adhesive surgical tape attached to a chain hanging from a pulley. The rats were suspended at a ~30° angle to the floor with only the forelimbs touching the floor. This allowed rats to freely move and access to food and water. The rats were anesthetized after 3 w of tail suspension. Bilateral femurs and tibiae were dissected and processed for micro-CT examination or total mRNA extraction. It is notable that the fat and connective tissues need to be stripped and bone marrow needs to be washed out when extract total mRNA for microarray test. All the experimental procedures were approved by the Committees of Animal Ethics and Experimental Safety of the Fourth Military Medical University (NO. 14022) and carried out in accordance with the approved guidelines.

### Micro-CT Analysis

The right femur of each rat was fixed in 4% paraformaldehyde for 24 h and then scanned using a micro-CT (Siemens, Germany). The scanning X-ray energy was set at 80 kv and 500 mA. The sample was scanned over a 360° rotation with an exposure time of 800 ms/frame at a resolution of 10.44 μm. The angle of increment around the sample was set to 0.5°. A 2.5×2.5×3 mm^3^ cube approximately 1.5 mm away from the proximal epiphyseal growth plate was selected as the Region of Interest (ROI) to display the microstructure of the femur. The Cobra software from the micro-CT was used to reconstruct the 2D projections into 3D. Several structural parameters were analyzed, including volumetric Bone Mineral Density (vBMD), relative Bone Volume (BV/TV), Trabecular Thickness (Tb.Th), Trabecular Number (Tb.N), Trabecular Separation (Tb.Sp) and Trabecular Pattern Factor (TPF)^59^.

### Primary Rat Osteoblasts Isolation and Culture

Primary rat osteoblasts (prOBs) were isolated as described previously^60^. Briefly, osteoblasts were derived from postnatal 24 h rat calvarias by sequential digestions for 30 min at 37 °C in 0.1% collagenase I (Sigma-Aldrich, Germany) and 0.25% trypsin (Sigma-Aldrich) mixture. Cells from the third and subsequent digests were collected and plated at 2 × 10^5^ cells/cm^2^ in Dulbecco’s modification of Eagle’s medium (DMEM) supplemented with 10% Fetal Bovine Serum (FBS). When cells was passaged for the first time, medium was switched to differentiation medium with 10% FBS containing 50 μg/ml ascorbic acid and 10 mM β–glycerophosphate to induce osteoblast differentiation and mineralization^61^. Cells were confirmed by the osteoblast phenotype characterized as the expression of Runx2 and ALP as well as the capacity to form mineralized bone nodules.

### Clinorotation to simulate microgravity

The Rotating Wall Vessel Bioreactor (RWVB) clinostat is an effective, ground-based tool that can be used to simulate a microgravity environment^62^,^63^. Weightlessness is achieved by maintaining cells in culture in a special vessel that rotates uniformly around a horizontal axis. There is a vector-averaged reduction in the apparent gravity acting on the cell while the vessel rotates 360 degrees. Under these conditions, cells are subjected to simulated microgravity conditions. A 2D-RWVB (developed by China Astronaut Research and Training Center, Beijing) was used in this experiment as previously described^64^. Briefly, prOB cells were seeded on coverslips at a density of 1×10^5^ cells and incubated for 24 h. The coverslips were then fixed in the vessel and placed 12.5 mm away from the rotational axis. The vessel was then completely filled with the culture medium. Gentle aspiration was performed to clear away air bubbles to avoid shear stress during rotation. Next, the vessels were fixed onto the clinostat and rotated around a horizontal axis at 30 rpm. The vessels rotating around a vertical axis were the control group. The entire system was placed in a humidified incubator at 37 °C under 5% CO_2_.

### miRNA Extraction and Microarray Profiling

Total mRNA was extracted using the mirVana™ miRNA Isolation Kit (Applied Biosystems, USA) according to the manufacturer’s protocol. RNA concentration was quantified using a NanoDrop 1000 Spectrophotometer (Thermo Scientific, USA). RNA quality was verified using an Agilent 2100 bioanalyzer (Agilent Technologies, USA) and measured using the RNA integrity number (RIN). RNA samples were discarded from further analysis if their RIN scores were <5.0.

miRNA microarray profiling was performed using Agilent Rat miRNA (8*15K) (Agilent Technologies) as previously described^65^. The microarray contains probes for 677 mature rat miRNAs found in the Sanger miRBase database^66^ version V16.0. Total RNA of bone tissues from CON (n = 3) and HU (n = 3) were subjected to microarray analysis. The microarray image information was converted into spot intensity values using Scanner Control Software Rev. 7.0 (Agilent Technologies). The signal after background subtraction was exported directly into the GeneSpring GX11.0 software (Agilent Technologies) for quantile normalization and further analysis. If the expression of an mRNA changed more than 2-fold in the HU group compared with the CON group, it was submitted to hierarchical clustering analysis.

### qRT-PCR Analysis

For qRT-PCR analysis, total RNA was extracted with TRIzol Reagent (Invitrogen, USA) according to the manufacturer′s protocol. The concentration and quality of total RNA were detected by measuring absorbance at 260 and 280 nm using a NanoDrop 1000 Spectrophotometer (Thermo Scientific).

For mRNA quantification, cDNA was prepared using the PrimeScript® RT reagent Kit (TakaRa Code: DRR037, Japan). Expression levels of target genes were determined quantitatively by an ABI 7500 realtime PCR system (Applied Biosystems) using SYBR^®^ Premix Ex Taq^TM^ II (TaKaRa Code: DRR820A) according to conventional protocols. The primers pairs were listed as follows: Runx2 (GenBank Accession NM_053470): F-5′- CCA TAA CGG TCT TCA CAA ATC C-3′ and R-5′-GCG GGA CAC CTA CTC TCA TAC T-3′; Osx (GenBank Accession NM_001037632): F-5′- CAG TAA TCT TCG TGC CAG ACC-3′ and R-5′-CTT CTT TGT GCC TCC TTT TCC-3′; ALP (GenBank Accession NM_013059): F-5′- AGA TGG ACA AGT TCC CCT TTG-3′ and R-5′-ACA CAA GTA GGC AGT GGC AGT-3′; Ep300 (GenBank Accession NM_013059): F-5′-GCT GCT CTC GGA CTA CCC TA-3′ and R-5′-GGC ACT CAT GTT GTT CAT GG-3′; GAPDH (NM_008084): F-5′-CAG TGC CAG CCT CGT CTC AT-3′ and R-5′-AGG GGC CAT CCA CAG TCT TC-3′.GAPDH was used as an internal control.

For miRNA quantification, PrimeScript® RT reagent Kit (TakaRa DRR037) was used again to synthesize the cDNA. But the component “Oligo dT Primer” and “Random 6 mers” were replaced with the bulge-loop miRNA RT primer designed by RiboBio (Guangzhou, China). The subsequent realtime PCR detection was the same as that of mRNA detection described above. U6 small nuclear RNA was used as an internal control.

### Transfection of Oligonucleotides

Primary rat osteoblast cells at 30–50% confluence cultured in by the DMEM normal serum media were shifted to serum-free OPTI-MEM media and transfected with oligonucleotides, including mimics and inhibitors of miR-132-3p (RiboBio), siRNA targeting Ep300 GenePharma (Shanghai, China) and their respective negative controls using the Lipofectamine 2000 reagent (Invitrogen) for 6 h according to the manufacturer’s instructions. Cells were then moved back to normal serum media. Normal or transfected cells were then subjected to clinorotation for 48 h and harvested for further analysis.

The siRNA targeted against Ep300 (siR-Ep300) were designed as follows: sense-5′-GCU ACU GCU GUG GCA GAA ATT-3′ and antisense-5′-UUU CUG CCA CAG CAG UAG CTT-3′. The negative control siRNA sequence was as follows: sense-5′-UUC UCC GAA CGU GUC ACG UTT-3′ and antisense-5′-ACG UGA CAC GUU CGG AGA ATT-3′. In each experiment, we optimized the amount of DNA, transfection reagents, and transfection duration to minimize toxicity and maximize efficiency. None of the transfection treatments exhibited noticeable effects on the rate of apoptosis.

### Immunoprecipitation (IP) Assays

The prOB cells were washed with ice-cold PBS and lysed in lysis buffer (1 mM EGTA, 150 mM NaCl, 1 mM EDTA, 20 mM Tris-HCl, and 1% Triton X-100, supplemented with a protease inhibitor cocktail, pH 7.4). The lysate supernatants were collected after centrifugation at 12000 g for 30 minutes at 4 °C. Parts of the supernatants were used for the detection of protein expression levels. The remaining lysates were used for immunoprecipitation with the appropriate primary and secondary antibodies and protein A/G (Santa Cruz, USA) or M2 (Sigma-Aldrich) beads. After incubating at 4 °C overnight, the beads were washed and the bound protein extracted by adding protein loading buffer and boiling. The separated proteins were then analyzed by western blots.

### Western Blot Analysis

Whole cell lysates and western blot analyses were performed as described previously^44^. The primary antibodies used were as follows: Runx2 rabbit monoclonal antibody (1:2000) (Epitomics, Burlingame CA), anti-Sp7/Osterix rabbit polyclonal antibody (1:5000) (Abcam, UK), anti-KAT3B/p300 antibody (1:2000) (Abcam), and anti-acetyl lysine antibody (1:1000) (Abcam). Membranes were incubated for 1 h at room temperature with the primary antibody in 5% milk followed by another incubation with a horseradish peroxidase-conjugated secondary antibody. The signals were detected using the Super Signal West substrate (Thermo Fisher Scientific, USA). Densitometry analyses of the western bands were performed using the Tanon Imaging software^67^.

### Alkaline Phosphatase Activity Assay

The prOB cells were seeded at 2 × 10^6^ cells/well in 6-well plates (Corning, NY) and cultured for 24 h before experimentation. To examine alkaline phosphatase activity, confluent cell layers were washed with PBS, lysed with 0.1 M M-PER mammalian protein extraction reagent (Pierce, USA) for 15 to 30 minutes, and finally centrifuged at 14,000 rpm for 15 minutes. The supernatants were then collected to determine their alkaline phosphatase activities using the alkaline phosphate (ALP) assay kit (Nanjing Jiancheng Technological Inc., Nanjing, Jiangsu, China). Protein concentrations were measured using the BCA Protein Assay Kit (Keygene, Shanghai, China). ALP activity (IU/L) was defined as the release of 1 nmol p-nitrophenol per minute per microgram of total cellular protein.

### Dual Luciferase Reporter Gene Construct

The fragment of the Ep300 3′UTR containing the predicted binding site for rno-miR-132-3p was amplified from rat genomic DNA. Amplicons were cloned and inserted into the XhoI/NotI cleavage sites of the PsiCHECK-2 vector (Promega, USA) downstream of the Renilla Luciferase reporter gene.

### Luciferase Assay

2T3 cells were selected for this assay based on their low endogenous expression of miRNAs. 2T3 cells were grown to 85–90% confluence in white 96-well plates in DMEM (Invitrogen) supplemented with 10% FBS, 1% nonessential amino acids, L-glutamine, and penicillin/streptomycin at 37 °C under 5% CO_2_. Cells were transfected with 20 ng empty PsiCHECK-2-vector, PsiCHECK-2-Ep300 3′ UTR, or PsiCHECK-2-MUT Ep300 3′ UTR for 4 h in reduced serum and antibiotic-free Opti-MEM with Lipofectamine 2000. Cells were co-transfected with the pre-miR-132, inhibitor or a negative control (miR control) (RiboBio) at a concentration of 20 nM, respectively. Firefly and Renilla luciferase were measured in cell lysates using a Dual-Luciferase Reporter Assay System (Promega) on a Fusion plate reader (PerkinElmer, USA). Firefly luciferase activity was used for normalization and as an internal control for transfection efficiency.

### miRNA Target Site Prediction

Computational miRNA target prediction analyses were performed using the databases TargetScan (http://www.targetscan.org/mmu_61/) and PicTar (http://pictar.mdc-berlin.de/.)^68^. The predicted genes were sorted based on the context scores, and those encoding osteogenic ossification-related extracellular signaling molecules were extracted. Sequence conservation was examined using University of California Santa Cruz genome browser (http://genome.ucsu.edu/).

### Statistical Analysis

Statistical analyses of the experimental data were conducted using SPSS 17.0 software. Data are expressed as the mean ± SD of at least three independent experiments. A one-way repeated measures analysis of variance (ANOVA) was used to compare the time course variables of miR-132-3p expression when exposed to simulated microgravity for different time periods (0 h, 24 h, 48 h, 72 h and 96 h). Statistical significance was tested using a two-tailed *t* test or a one-way ANOVA, and a *p* value less than 0.05 was considered to be significant.

## Additional Information

**How to cite this article**: Hu, Z. *et al.* miRNA-132-3p inhibits osteoblast differentiation by targeting Ep300 in simulated microgravity. *Sci. Rep.* **5**, 18655; doi: 10.1038/srep18655 (2015).
